# Applications of AI in multi-modal imaging for cardiovascular disease

**DOI:** 10.3389/fradi.2023.1294068

**Published:** 2024-01-12

**Authors:** Marko Milosevic, Qingchu Jin, Akarsh Singh, Saeed Amal

**Affiliations:** ^1^ Roux Institute, Northeastern University, Portland, ME, United States; ^2^ College of Engineering, Northeastern University, Boston, MA, United States

**Keywords:** multi-modal data, clinical imaging, cardiovascular, cardiac, segmentation, registration, fusion

## Abstract

Data for healthcare is diverse and includes many different modalities. Traditional approaches to Artificial Intelligence for cardiovascular disease were typically limited to single modalities. With the proliferation of diverse datasets and new methods in AI, we are now able to integrate different modalities, such as magnetic resonance scans, computerized tomography scans, echocardiography, x-rays, and electronic health records. In this paper, we review research from the last 5 years in applications of AI to multi-modal imaging. There have been many promising results in registration, segmentation, and fusion of different magnetic resonance imaging modalities with each other and computer tomography scans, but there are still many challenges that need to be addressed. Only a few papers have addressed modalities such as x-ray, echocardiography, or non-imaging modalities. As for prediction or classification tasks, there have only been a couple of papers that use multiple modalities in the cardiovascular domain. Furthermore, no models have been implemented or tested in real world cardiovascular clinical settings.

## Introduction

Cardiovascular diseases (CVD) are the worldwide leading cause of death, representing 32% of global deaths (1). For patients suffering from cardiovascular diseases in the United States from 2000 to 2008, the mean annual direct medical costs was $18,953, which extrapolates to over $400 billion for the entire nation (2). In 2016, the American Heart Association found that 41.5% of Americans had at least one CVD condition, and they projected that costs would exceed $1.1 trillion dollars by 2035 (3). It is estimated that 5%–10% of US healthcare spending could be saved with wider adoption of artificial intelligence technologies (4).

Many different imaging technologies are used in cardiac assessment: x-ray, computed tomography (CT), multiple varieties of magnetic resonance imaging (MRI), and echocardiography (Echo). Physicians consult multiple of these modalities, along with lab results, vital signs, and other clinical observations. While most research in artificial intelligence for healthcare has been on a single modality, as the field progresses, there have been increasing attempts to leverage multiple modalities for a variety of tasks.

**Figure 1:**
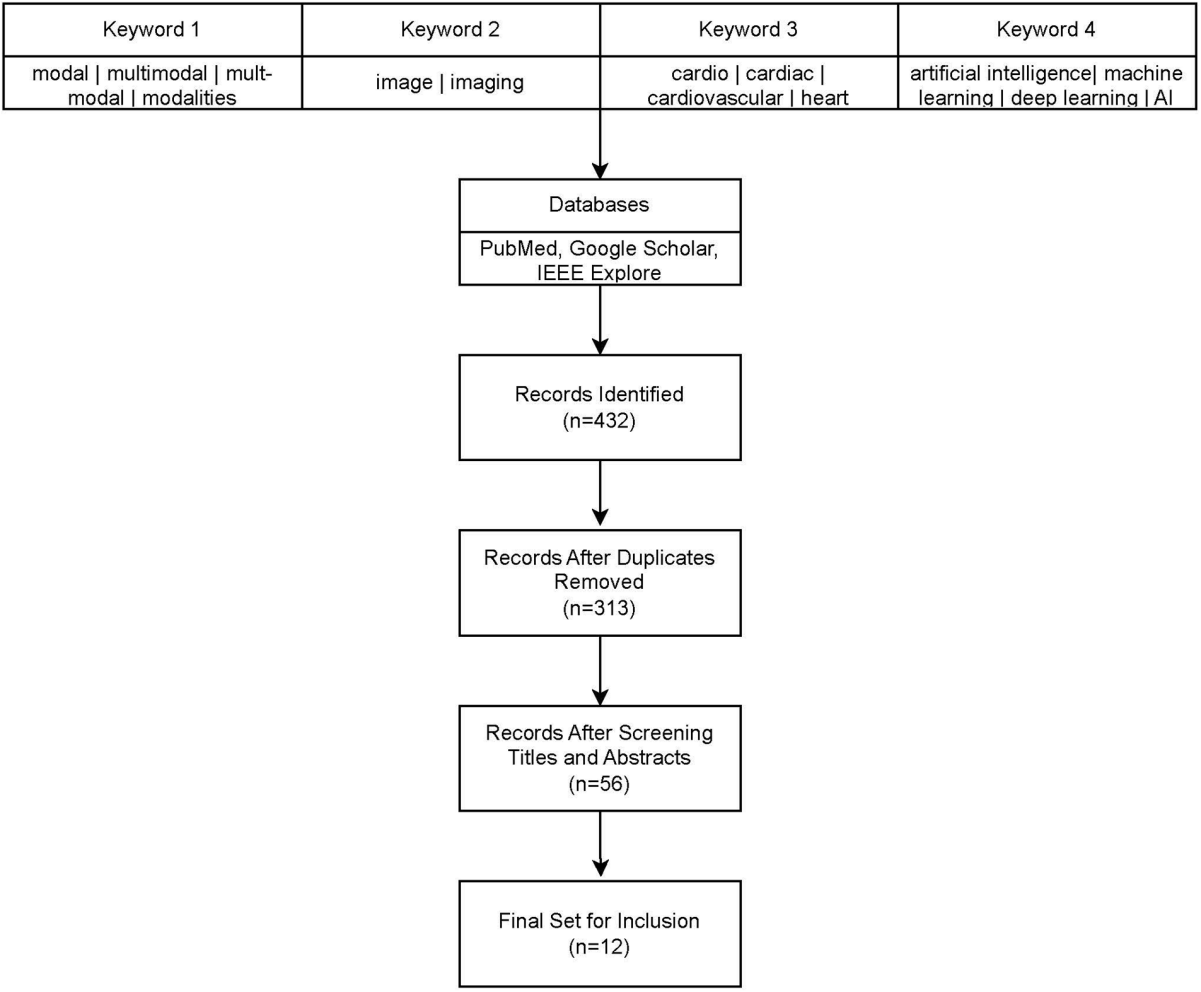
Literature review and filtering flowchart.

There have been several recent survey articles on multi-modality for cardiovascular diseases with different focuses (5–9). In this survey, we focus on providing a near comprehensive review of multi-modal imaging for cardiovascular diseases since 2018. We retrieved all articles from PubMed, Google Scholar, and IEEE-Xplore that contained terms relevant to multi-modality, cardiac systems, and artificial intelligence. See Figure 1 for a flowchart of our literature search and selection process. We eliminated any papers whose authors did not detail the architecture or used commercial technology in their models, such as Siemen's TrueFusion or Philip's EchoNavigator. With the publication of three open datasets for competition by Zheng, there have been many recent papers published using the same datasets for similar problems (10–12). For these papers, we tried to choose the most representative papers for each task.

Most recently published papers addressed registration, segmentation, and fusion of multi-modal images. Registration is the problem of transforming two or more pictures of the same objects to ensure they are aligned with each other (13). In the context of medical images, it is important that all anatomical structures are aligned across the images. Image fusion is generally the process of combining two or more images into the same space or combined image, whereas image segmentation is the process of registering regions of interest in an image. We examined nine papers about registration, segmentation, and fusion in Section 2.1, but could only identify two papers addressing predictive tasks or for diagnostic aids discussed in Section 2.2. Moving beyond cardiovascular imaging, multi-modal imaging extends its reach, prompting us to highlight two papers of interest in Section 2.3. These papers, although outside the cardiovascular domain, may serve as inspirations for future directions. While promising results have emerged in the registration, segmentation, and fusion of various magnetic resonance imaging modes with both each other and computer tomography scans, there remains a gap in the literature concerning modalities such as x-ray, echocardiography, or non-imaging modalities like electronic health care data. Additionally, the exploration of multi-modal imaging for cardiovascular prognosis or diagnostic assistance is relatively limited. Moreover, there is a noticeable absence of investigations applying multi-modal imaging to real-world cardiovascular clinical scenarios.

## Literature review

### Segmentation, fusion and registration in cardiovascular imaging

Wang, et al. propose a fusion segmentation model for segmenting aortas (14). Skip connections in a neural network are connections that connect two non-adjacent layers in an architecture to allow for models to retain information from higher layers (15). Encoder-Decoder networks are a broad category of models that first encodes a structure into a representation and then decodes the representation into another structures (16). The authors employ an encoder-decoder convolutional network based on U-Net to minimize cross-entropy pixel-wise loss for both Computed Tomography (CT) and Magnetic Resonance (MR) scans with skip connections between layers. In between the encoding and decoding layers, they include a fusion layer of the encoded representations of both CT and MR scans which produces segmentations of both the CT and MR scans that includes information from the other (17). The model is trained and tested on a dataset with CT and MR scans of 21 participants diagnosed with abdominal aortic aneurysms. Unfortunately, the authors do not provide any summary metrics for the performance of their model on their test set, but they do note that their validation accuracy in their training for CT separately is 99.1% compared to 98.8% for fusion-CT. They also note that rotation of the scans induces an increase in feature distance of the fusion models which was not observed in the separately trained models.

Peoples, et al. propose a registration method for transesophageal echocardiography (TEE) to a preoperative cardiac CT scan in order to aid navigation of endoscopy (18). The authors construct a complicated nonrigid registration technique where the four cardiac chambers are manually segmented in the preoperative CT and chambers are manually delineated in the perioperative CT. The four cardiac chambers are treated as separate structures. For each TEE ultrasound image, a point set is extracted using an edge detector and an orientation is assigned. Registration of the TEE images to the matching 3D slices of the CT scan are modeled using a hybrid mixture model and expectation maximization to match the TEE images to the 3D CT scans. The data set consisted of 4 patients with a total of 27,000 slices. With such a small sample size of patients, they found no statistically significant difference for the root mean square error between the ultrasound virtual points and model points and the expected root mean square error. The authors claim this study is proof of potential feasibility of the model.

Zhuang introduces a method to simultaneously segment multi-source images in a common space by using multivariate mixture models (MvMM) and a maximum log-likelihood and tests it on segmentation of scans of myocardial tissues (10). The Multivariate Mixture Model is a generalization of Gaussian Mixture Models that accounts for multiple modality image vectors. Once the tissue type of a position is known, the intensity distributions of different images become independent. Consequently, the probability that a vector of images will be produced from given parameters becomes a product of the probabilities per image, and the intensity probability density function per image is then the standard multi-component Gaussian Mixture Model. The MvMM is initialized and regularized by the prior probabilities from an atlas which can be registered to the common space of the target images using the conventional methods in the atlas-based segmentation framework (18). To account for spatial and anatomical constraints, Zhuang incorporates a Markov Random Field to model neighborhood dependencies for each pixel. Since there exist potential image misalignment and variance in pixel-dimensions, transformations on both slices of an image and between images were modelled. To evaluate the model, cardiac magnetic resonance (CMR) sequences were collected from 45 patients. Each of these patients were scanned using a late gadolinium enhancement (LGE) CMR, T2-weighted CMR, and balanced-Steady State Free Precession (bSSFP) cine sequence. These three scans capture different structures of the heart. To evaluate the validity of the segmentation results, the Dice metric, average contour distance, and Hausdorff distance were calculated between the automatic segmentation and the corresponding gold standard segmentation. Since Zhuang evaluated the model across a wide variety of metrics, scans, and configurations, it is not feasible to report all of them, but r -efer to Table 1 for performance of the standard model configuration. In LGE scans, the proposed model outperformed a conventional GMM model (*p* < 0.01), and although U-Net obtained a similar average Dice score as the proposed model, it had twice the standard deviation.

**Table 1:** Performance evaluation of model by scan and structure.

	Dice			ACD (mm)		Hausdorff distance (mm)	
	Endocardium	Epicardium	Myocardium	Endocardium	Epicardium	Endocardium	Epicardium
LGE	0.866 ± 0.063	0.896 ± 0.036	0.717 ± 0.076	2.54 ± 1.00	2.62 ± 0.91	10.6 ± 4.67	11.2 ± 4.06
T2	0.794 ± 0.124	0.908 ± 0.043	0.717 ± 0.129	3.75 ± 2.18	2.46 ± 1.27	11.9 ± 5.90	9.94 ± 5.94
bSSFP	0.903 ± 0.048	0.917 ± 0.027	0.764 ± 0.064	2.06 ± 0.96	2.16 ± 0.81	9.23 ± 5.06	10.7 ± 4.56
T2 (+GMM)	0.827 ± 0.094	0.878 ± 0.046	0.744 ± 0.094	2.88 ± 1.69	2.46 ± 1.13	10.6 ± 5.99	12.1 ± 5.47

Blendowski, et al. propose a modality independent convolutional encoder-decoder network mapping to a common shape space (19). Their model is then used to align computer tomography (CT) and magnetic resonance imaging (MRI) scans. Early attempts in combining different modalities, such as by Zöllei et al., had misleading statistical correlations in image patterns that did not correspond to real anatomical structures (20). Blendowski et al. do not require aligned images or ground-truth deformation fields to be trained. To accomplish these tasks, first a convolutional auto-encoder with no skip-connections is used to generate segmentation of the different modalities. For the training, a joint-training of both the CT and MRI images as well as the segmentations is used following previous work of Bouteldja, et al. (21). Second, they seek to align the CT and MRI scans through iteratively guided registration on their reconstructed shapes by using gradient descent to minimize cross-entropy loss of a linear interpolation between the two encodings. To test their model for segmentation, the authors use a dataset of 20 MRI and 20 CT whole-heart images with substructures. With a four-fold cross-validation, they achieve Dice–Sørensen coefficient of 0.84 for CT and 0.79 for MRI. This fell slightly short of the U-Net segmentation, which achieved scores of 0.87 for CT and 0.84 for MRI on the same dataset. Nevertheless, the CAE-generated segmentations can play a crucial role in guiding the iterative registration of multimodal segmentation. Their method produced a Dice–Sørensen coefficient of 0.653 compared to 0.608 from classical self-similarity composition methods by Heinrich et al. (22).

Zheng, et al. develop a deep learning multi-modal framework for Cardiac MR (CMR) image segmentation using three different CMR scans: late gadolinium enhancement (LGE), T2-weighted (T2), and the balanced-Steady State Free Precision (bSSFP) cine sequence (23). The three types of CMR have different advantages, with LGE enabling clear observation of myocardial infarction, T2 showing local acute injury, and bSSFP can capture cardiac motion and clear boundaries (24). The first step of the model is to perform automatic registration of the T2 scan onto the bSSFP scan using the Normalized Mutual Information criterion (25). After co-registration of the scans, the second step is to feed the two images into U-Net to generate a segmentation of the bSSFP scan. The next step was to register the bSSFP and T2 scans to the LGE scan, as well as to appropriately transform the generated segmentation labels to LGE space. Afterwards, all three co-registered scans were once again inputted into a U-Net network to segment the LGE space using the generated bSSFP segmentation labels as a ground truth. Finally, the model was fine-tuned using 5 LGE scans with true segmentation. The dataset consisted of 45 patients with LGE, T2, and bSSFP CMR scans from the dataset released by Zhuang as part of a challenge (10). The model was evaluated on its ability to segment three different structures, achieving Dice coefficients of 0.8541 ± 0.0581 for the left ventricular, 0.7131 ± 0.1001 for the left ventricular myocardium, and 0.7924 ± 0.0871 for right ventricular (RV). Since the test set of the competition dataset was not released at the time of publication, the authors were not able to compare their results to other models.

Chartsias, et al. introduce DAFNet, a multi-component 2D model for multimodal and semi-supervised segmentation, specifically for myocardial LGE scans and cine-MR scans (26). DAFNet seeks to map multimodal images of an object into disentangled anatomy and modality factors, and then fuses the disentangled anatomy factors to combine multimodal information. Specifically, a U-Net based encoder and decoder structure is used to disentangle and create the segmentation, and a spatial transformer network is used to fuse the anatomical structures before being decoded using either a FiLM-based decoder or SPADE-based decoder (17, 27, 28). They evaluate their models on three different datasets, one of which is a set of 28 patients with cine-MR and LGE MR scans (29), and another is a set of cine-MR and CP-BOLD images of 10 mechanically ventilated canines (30). DAFNet was compared in multiple configurations to various baseline and benchmark models at varying levels of annotations. When all target annotations are available and segmenting the target modality, the usage of multiple inputs at inference time by DAFNet obtains similar or better Dice score than all other benchmarks, but considerably reduces the standard deviation. When target annotations are not all available, DAFNet significantly outperforms all other models at unsupervised learning. Similarly, DAFNet outperforms in semi-supervised cases as well.

Ding, et al. propose a multi-modality registration network MMRegNet to align medical images to a common space (31). MMRegNet is constructed on a U-shape convolutional network which takes a pair of images as input and predicts forward and backward dense displacement fields built on previous work from the authors (32). The authors evaluated their model on a set of 20 MR and 20 CT images for left ventricle registration from a public dataset (33). MMRegNet was trained to perform registration of MR to CT images and evaluated on Dice Coefficient Score and Average Surface Distance between the corresponding label of moved and fixed images. MMRegNet was compared to three classical and state-of-the-art registration methods Sy-NCC (34), Sy-MI (34), and VM-NCC (30). MMRegNet outperformed all three with a DSC of 80.28 ± 7.22, and only VM-NCC had a better ASD score.

Luo and Zhuang construct an information-theoretic metric called the χ-metric and co-registration algorithm χ-CoReg that identifies the statistical dependency between an arbitrary number of images (35). They combine this with a deep learning network to allow for end-to-end simultaneous registration and segmentation of medical images across modalities. The authors follow a similar probabilistic framework to that of Zhuang's previous work described above (10). Given a set of images, co-registration aims to find the corresponding set of transformations that aligns them into a common coordinate system. The issue with classical information theoretic approaches to co-registration is that it becomes increasingly difficult to compute the joint entropy as the size of the set of images increases. By assuming an *a priori* knowledge of common anatomy across the set of images as a set of latent variables, the authors reduce the uncertainty of the intensity-class mutual information metric, and they then define the χ-metric as the sum of the intensity class mutual information metric and the Kullback–Leibler divergence between the joint distribution and the product of its marginals, which eliminates the computation of the joint entropy term. χ-CoReg is classical optimization of the χ-metric across spatial transformations and common space parameters. The deep learning network architecture is built with an encoder, a bottleneck, a segmentation decoder and a registration decoder that consists of residual convolutional blocks. The authors evaluate their model across a variety of datasets and metrics, one of which is the MoCo dataset which consists of mid-ventricular short-axis first-pass cardiac perfusion for 10 patients at both rest and adenosine induced stress phases. The χ-CoReg outperformed all other comparable co-registration methods across different transformations, with Dice similarity coefficient (DSC) scores of 78.4 ± 8.7 for translations, 79.2 ± 7.2 for rigid transformations, and 80.1 ± 6.0 for FFD transformations. The authors also tested the algorithm for segmentation on expanded version of Zhuang's previous dataset with 45 patients with LGE CMR, T2-weighted CMR, and bSSFP. The proposed model outperforms the MvMM models with segmentation Dice coefficient of 92.6 ± 2.0 for LGE, 92.7 ± 3.4 for T2, and 92.4 ± 3.1 for bSSFP.

There are a variety of sophisticated methods and architectures employed for image registration. Image segmentation seems to be narrowly dominated by U-Net architectures (17), with the exception of Zhuang who employs a multi-variate mixture model and Blendowski who employs a general encoder-decoder architecture without skip-connections (10, 19). Segmentation performed well for cardiovascular tasks, with Dice coefficient scores of greater than 80 for most tasks. There have not been any papers that assessed image fusion in and of itself for cardiovascular systems, rather fusion was generally employed to aid in segmentation.

### Prediction and diagnostic aid for cardiovascular diseases

Chaves, et al. develop a framework that leverages deep learning and machine learning models for opportune risk assessment of ischemic heart disease (IHD) (36). Ischemic heart disease, or coronary heart disease, are heart problems caused by narrowed coronary arteries, and is the leading cause of death in both men and women—causing one of every six deaths in the US (37). Traditional diagnostic tools such as Framingham coronary heart disease risk score (FRS) and pooled cohort equations (PCE) typically use demographic factors, cholesterol values, and blood pressure, but only have modest performance with c-statistic values of between 0.66–0.76 for FRS and 0.68–0.76 for PCE (38). The proposed model utilizes automatically measured imaging features extracted from abdominopelvic CT examinations, along with relevant information from the patient's electronic medical records (EMR). Their dataset consisted of 8,197 CT images from patients with at least 1 year of follow-up and 1,762 CT images from 1,686 patients with at least 5 years of follow-up. All available EMR data dated before the scans was collected for each patient.

Three baseline models were constructed. First, an imaging only CNN model that was based on EfficientNet-B6 was trained to predict the risk of ischemic heart disease using a single axial CT slice (39). The initial model weights were derived from a EfficientNet-B6 model that was pre-trained on ImageNet classification (40). The second was a segmentation model built using a 2.5-dimension U-Net CNN that was trained on a set of 320 axial CT slices manually segmented (17). For each segmented CT slice, two body composition imaging biomarkers were calculated: ratio of visceral to subcutaneous adipose tissue (VAT/SAT ratio) and average muscle radiodensity in Hounsfield units. These two metrics were used as features for a L2 logistic regression with ten-fold cross validation to predict IHD outcomes at 1 and 5 years. The third baseline model was limited to only clinical records. A variety of vital signs, demographic data, relevant laboratory results, medications, and were compiled. In total 434 features were extracted and used for XGBoost to predict ischemic heart disease.

**Figure 2:**
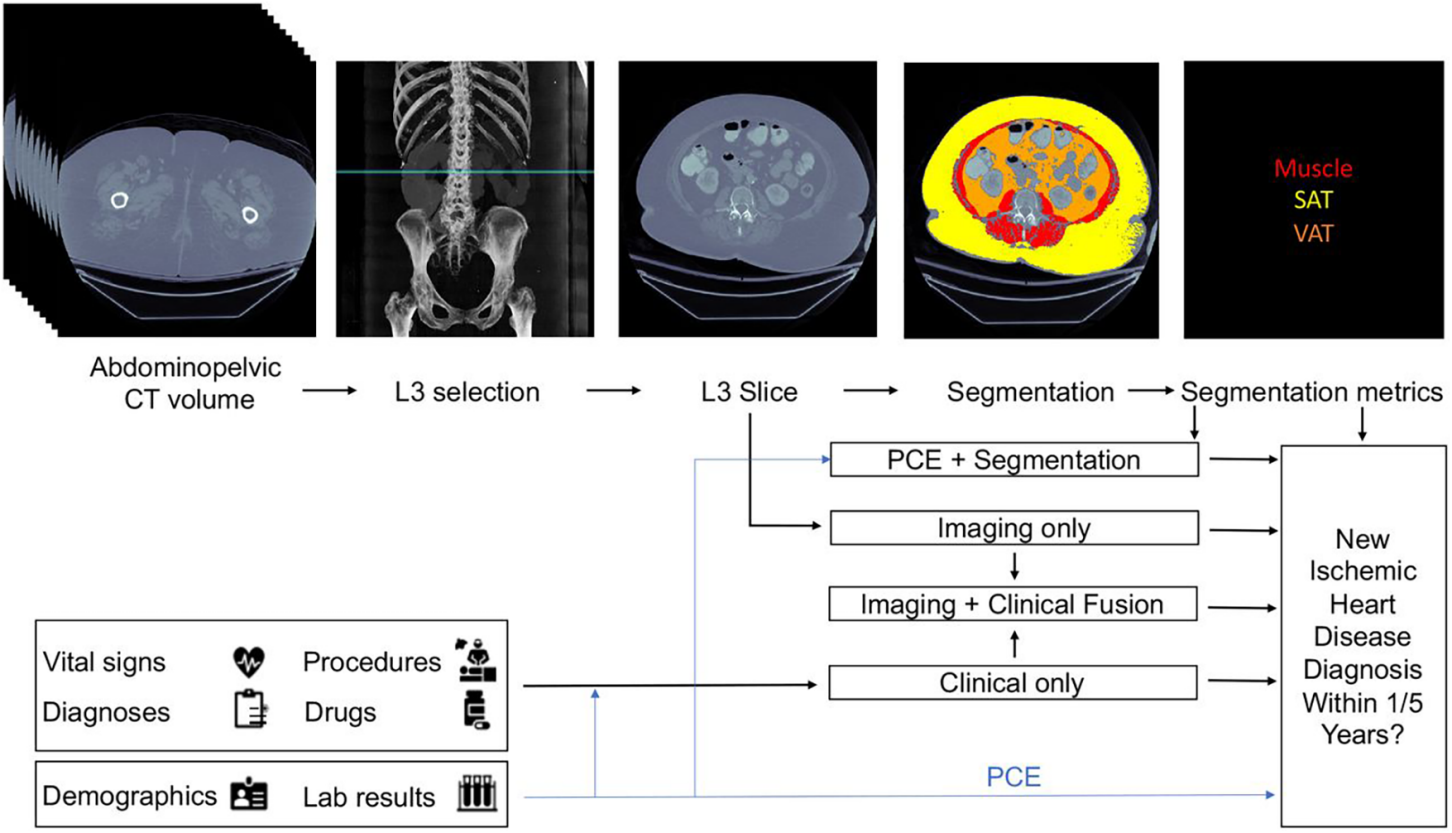
Proposed architecture of multi-modal data fusion combining imaging and clinical data. The blue line shows which sources are used by the Pooled Cohort Equations (PCE). Figure reprinted from Chaves, et al. (36).

From these three baseline models, three fusion models were created. The first fusion model combined pooled cohort equations with the segmentation model (PCE + Segmentation) by concatenating the PCE features with the VAT/SAT and average muscle radiodensity generated by the segmentation model. The second fusion model combined the risk output of the imaging model with the risk output from the clinical model by using an L2 logistic regression (Imaging + Clinical). The final fusion model (Figure 2) combined the risk factors from imaging, clinical and segmentation.

All six models were compared to each other and to the classic risk factors given by FRS and PCE. All models were evaluated on both the area under curve of the receiver operator curves (AUCROC) and precision recall curves (AUCPR), and 95% confidence intervals were obtained by the stratified bootstrap method. Refer to Table 2 for a full report of statistics. In the 1-year cohort, none of the models exhibited statistically significant performance surpassing PCE. in the 5-year cohort, both the clinical and imaging baselines, as well as the fusion approaches involving imaging, clinical, and segmentation, significantly outperformed PCE. The primary limitation of this approach is that modalities are separately modelled before fusion, thus it may miss the full range of interactions between modalities.

**Table 2:** Proposed model performances measured by AUCROC and AUCPR.

Model	1y AUROC (95% CI)	*P*	1y AUCPR (95% CI)	*P*	5y AUROC (95% CI)	*P*	5y AUCPR (95% CI)	*P*
FRS	.71 (.67–.76)	.**04**	.09 (.07–.12)	.06	.71 (.66–.76)	.24	.40 (.35–.48)	.73
PCE	.75 (.71–.81)	–	.12 (.10–.17)	–	.73 (.69–.78)	–	.41 (.36–.48)	–
Segmentation	.70 (.65–.74)	.10	.08 (.07–.10)	.08	.73 (.68–.78)	.85	.43 (.38–.51)	.64
PCE + Segmentation	.76 (.71–.81)	.68	.12 (.10–.15)	.90	.74 (.70–.79)	.45	.43 (.38–.51)	.41
Clinical only	.76 (.72–.81)	.57	.12 (.10–.17)	.98	.84 (.80–.87)	**<**.**001**	.64 (.58–.72)	**<**.**001**
Imaging only	.74 (.70–.78)	.70	.10 (.08–.15)	.55	.81 (.76–.85)	.**02**	.64 (.57–.71)	**<**.**001**
Imaging + clinical fusion	**.77** (**.73**–**.81)**	.38	**.13** (**.10**–**.19)**	.73	**.86** (**.82**–**.90)**	**<**.**001**	**.70** (**.63**–**.77)**	**<**.**001**
Imaging + clinical + segmentation fusion	.74 (.70–.79)	.74	**.13** (**.10**–**.18)**	.78	**.86** (**.82**–**.89)**	**<**.**001**	**.70** (**.63**–**.77)**	**<**.**001**

*P*-values correspond to comparisons with PCE. Largest AUC values and *P*-values less than 0.05 are bolded.

Myocardial infarction (MI) due to prolonged ischemia in the heart can lead to the development of myocardial scarring, which is a common diagnostic marker for intervention. Guo, et al. develop an automated model for quantifying the heterogeneity in myocardial tissue from 2D short-axis cine and 3D LGE MRI scans (41). The first step of the model was to take the cine slices to interpolate a three-dimensional image and create a single segmentation using U-Net that was validated with the STAPLE algorithm (42). The second step of the model was to register the interpolated cine images to the 3D LGE scans using an affine registration that used block matching (43). The resulting transformation was used to register the cine segmentation to the LGE scans and constrain the heterogeneity analysis to the area within the segmentation. The LGE image signal intensities were clustered into 3 classes using classic k-means clustering. The largest connected component of the class with lowest intensity was used to identify an initial remote region. Regions of gray zone and infarct core were identified by either using a standard-deviation threshold (SD) method or full-width-at-half-maximum clustering (FWHM) method (44). Finally, the resulting areas were cleaned for noise by using a normalized cut method (45).

To evaluate the model, ten pigs (Yorkshire swine) were scanned using both balanced-Steady State Free Precision (bSSFP) for generating 2D short-axis cine MRI scans and late gadolinium enhancement (LGE) 3D MRI scans. For the 87 cine slices that contained scar tissue, the segmentation achieved a Dice similarity coefficient of 0.87 ± 0.12. The registration of the cine interpolations to LGE scans had a dice similarity coefficient of 0.90 ± 0.06. The validate the quantification, two observers manually segmented the LGE scans for gray zone (GZ), infarct core (IC), and healthy myocardium. For both the SD and FWHM methods, automated IC, GZ, and IC + GZ volumes were strongly correlated with manual measurements with the Pearson correlation being greater than 0.70 across all cases. The correlations could not be statistically distinguished from interobserver correlations with *p*-value of 0.13.

In the past 5 years, we identified only two papers that employed multi-modal imaging for tasks beyond registration, segmentation, and fusion with open data. Chaves, et al. had the only paper to combine electronic health care record data with imaging (36). Guo, et al. essentially adapt a segmentation task to calculate an important diagnostic metric (41).

### Beyond cardiovascular

**Figure 3:**
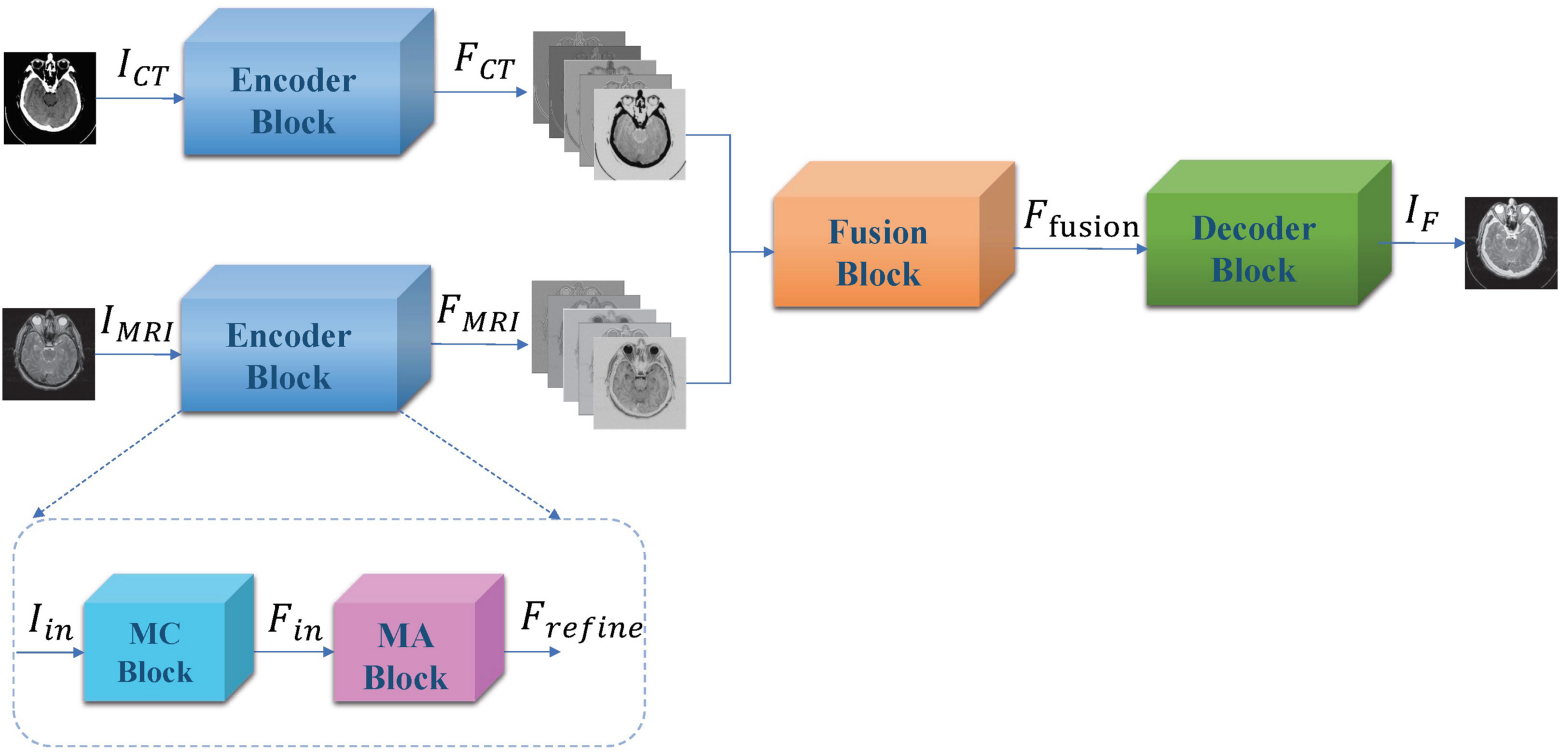
Overall framework of proposed muti-scale mixed attention network. Figure reprinted from Liu, et al. (46).

Liu, et al. propose a convolutional based CT and MRI image fusion model MMAN (46). The model consists of three parts: two separate encoder blocks for each modality (CT and MRI), a fusion block and a decoder block (Figure 3). The encoder blocks both consist of two sub-networks, the first a multi-scale convolutional (MC) block and the second a mixed attention (MA) block. The MC block is inspired by Res2net (47), a four branch network to extract features at various depths. The MA block consists of a dual channel attention module and a dual channel spatial attention module. The decoder block is a straightforward stack of convolutional layers to recover the fused image from the fused features. The dataset was evaluated on 561 pairs of CT and MRI images from the Whole Brain Atlas. The authors compare their fusion model to 7 standard and state-of-the-art fusion models across 6 different metrics: correlation coefficient (CC), mutual information (MI), nonlinear correlation information entropy (NCIE) (48), spatial frequency (SF), phase congruency (PC) (49), and the sum of the correlations of differences (SFD) (50). The proposed fusion model scored 0.8179 CC, 4.2488 MI, 7.8951 SF, 0.3882 PC, 0.8124 NCIE, and 1.6040 SCD, outperforming all other models across all metrics except phase congruency. MRI images provide high resolution anatomical information for soft tissues, while CT images can detect dense structures, thus the fusion of such images can hopefully provide the benefits of both scans and aid physicians in more efficient diagnosis. The paper proposes an interesting architecture that should easily be transferable across domains.

**Figure 4:**
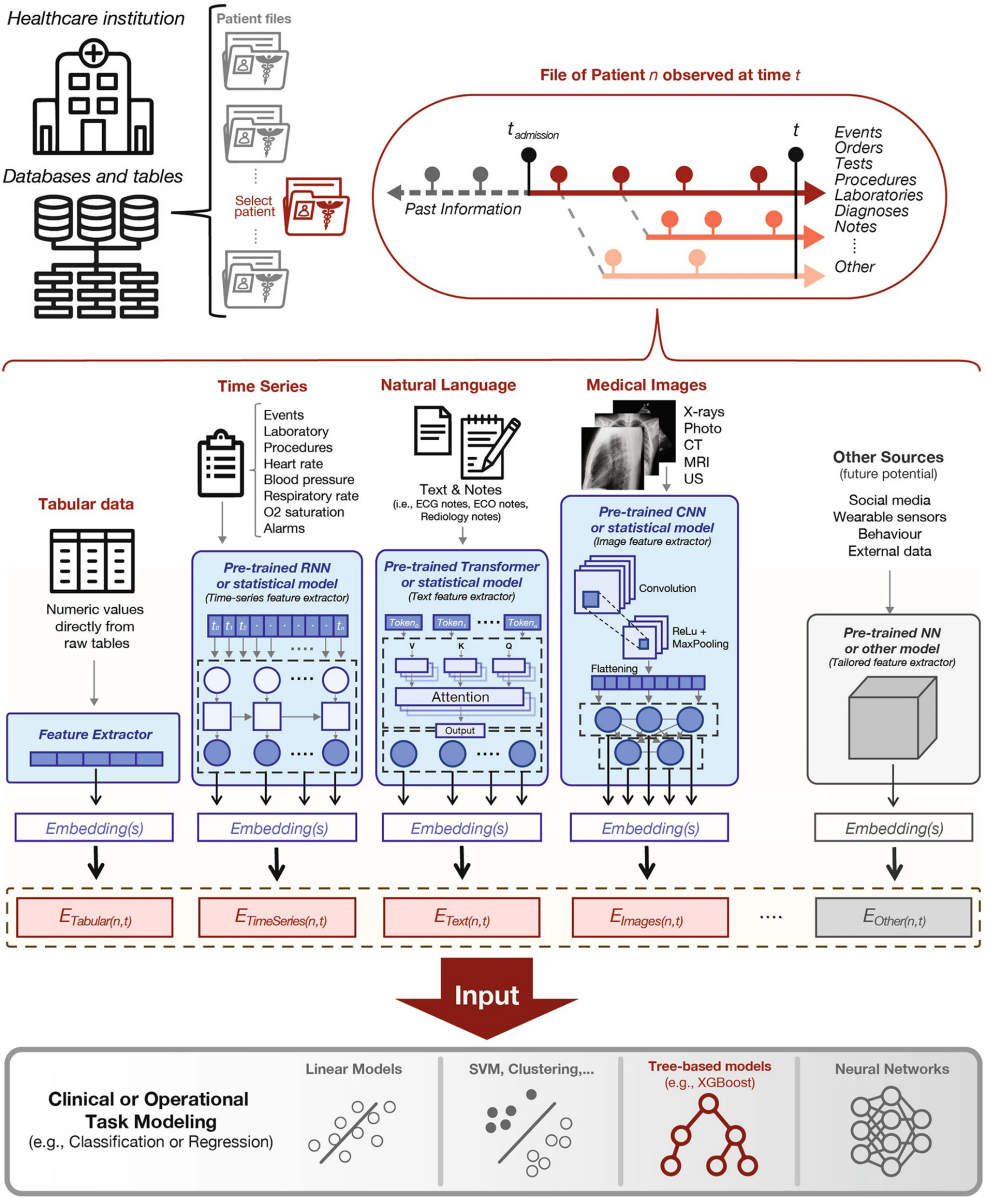
Proposed holistic artificial intelligence in medicine (HAIM) framework. Figure reprinted from Soenksen, et al. (51).

Soenksen, et al. propose a unified Holistic AI in Medicine (HAIM) framework to test large multimodal health databases across a variety of predictive tasks (51). They test their framework on a large dataset with 6,485 patients and 34,537 entries across four different modalities: tabular, time-series, text, and x-ray images. Their HAIM framework (Figure 4) consists of creating embeddings for the four different modalities and then fusing the embeddings with XGBoost (52). Tabular data was transformed and normalized as appropriate, time-series were embedded by generating representative statistical metrics, natural language inputs were processed by a pre-trained transformer to generate an embedding of fixed size, and x-ray images were processed using a pre-trained CNN network. HAIM was evaluated across 12 predictive tasks: length of stay, 48 h mortality, fracture, lung lesion, enlarged cardio mediastinum, consolidation, pneumonia, atelectasis, opacity, pneumothorax, edema, and cardiomegaly. HAIM outperformed canonical single-modality approaches for all 12 tasks by an average percent improvement of 9%–28% of the area under the receive operator curve. HAIM is a framework that can be straightforwardly expanded with other modalities and other tasks.

We included Liu, et al. work since they introduce a novel architecture that should be easily adaptable to cardiovascular imaging, and evaluate their model purely on image fusion itself (46). Soenksen, et al. provide a framework on how large health record datasets combined with imaging information can be used for a variety of predictive tasks with a simple joining of architectures (51).

## Discussion: limitations and future directions

The recent publication of the three open multi-modal datasets has led to a lot of novel research, which shows that research in AI for healthcare is often driven by the dataset available. Refer to Table 3 for the papers included in the review. Regrettably, these open multimodal datasets are constrained both in size and modality scope. In particular, the scarcity of open multi-modal datasets with labeled pathologies contributes to the comparatively few published papers on the diagnosis or prediction of cardiovascular diseases and conditions. Specifically, we aim to adapt Ghanzouri et al. methodology for diagnosing peripheral artery disease by integrating electronic health record information and merging it with imaging data (51). While most of the papers have explored modal fusion involving various magnetic resonance imaging and computer tomography scans, the integration of modalities like x-ray, echocardiography, and non-imaging modalities remains relatively scarce. Even beyond our constraint to open datasets, our search identified only one paper in the last 5 years that combines echocardiography and magnetic resonance (52).

**Table 3:** Summary of reviewed papers categorized by section and arranged chronologically, detailing modalities, objectives, and architectures.

Authors	Year	Modalities	Objectives	Architectures
Wang et al. (14)	2018	CT, MRI	Aorta segmentation	U-Net
Peoples et al. (18)	2019	Transesophageal echocardiogram, CT	Registration	Hybrid mixture model
Zhuang (10)	2019	LGE, T2, and bSSFP MRI	Registration, segmentation	Mixture models, Markov random field
Blendowski et al. (19)	2020	CT, MRI	Segmentation	Encoder-decoder
Zheng et al. (23)	2020	LGE, T2, and bSSFP MRI	Registration, segmentation	U-Net
Chartsias et al. (26)	2021	LGE and bSSFP MRI	Fusion, segmentation	U-Net, SPADE, FiLM
Ding, et al. (31)	2022	CT, MRI	Registration	U-shape CNN
Wang, et al. (53)	2022	LGE, T2, and bSSFP MRI	Myocardial scar and edema segmentation	U-Net, deep auto-weighted supervision, pixelwise attention modules
Luo and Zheng (35)	2023	LGE, T2, and bSSFP MRI	Registration, segmentation	χ-CoReg, encoder-decoder
Chaves et al. (36)	2021	CT, EHR	Ischemic heart disease diagnosis	EfficientNet-B6, XGBoost, logistic regression
Guo, et al. (41)	2021	bSSFP and LGE MRI	Segmentation, myocardial tissue heterogeneity quantification	U-Net, STAPLE, K-means, Full-Width-At-Half-Maximum Clustering
Liu, et al. (46)	2022	CT, MRI	Fusion	Encoder-Decoder, Res2Net, Dual Attention
Soenksen et al. (51)	2022	x-Ray, tabular data, time-series, and EHR	Various predictive tasks	CNN, Transformer, XGBoost

For registration and fusion, a diverse range of methods and models are utilized across the surveyed literature. In contrast, variations or modifications of U-Net seem to be the near universal favorite for segmentation tasks. There have been many recent papers on using generative adversarial networks (GANs) to segment medical images (54). In future, we anticipate increased experimentation with GANs for the segmentation of multi-modal images, and possibly even automatic annotation of such images.

One of the primary directions of current AI is utilizing pretrained foundation models such as BERT (54). Although foundation models have demonstrated success in other domains, task-specific models have generally proven more effective for real-world medical imaging analysis (53). Our review reveals that only the architecture proposed by Chaves, et al. effectively incorporates foundation models, underscoring the potential for further research in this direction (30).

In a real-world clinical setting, the robustness of models holds significant importance. Many models were trained exclusively on data from a single source with curated scans, making it challenging to ascertain their generalizability. As more diverse datasets become accessible, it becomes imperative to assess the performance of these models across varied datasets. Additionally, addressing the challenge of missing modalities remains a gap in the scope of clinical applications, with few models addressing how to account for missing modalities. Notably, none of the models in the reviewed papers underwent evaluation by experts for interpretability and usability in hypothetical real clinical workflows, as demonstrated in Singh et al. (56).

Over the last 5 years there has been a considerable amount of work in artificial intelligence that leverages multi-modal imaging. The successful application of AI in this context has the potential to significantly impact clinical decision-making, ultimately resulting in improved patient outcomes and a reduction in healthcare costs.
